# Alcohol consumption and mortality from four alcohol-related cancers in Australia 1950-2018: a time series analysis

**DOI:** 10.1038/s41416-025-03273-1

**Published:** 2026-01-09

**Authors:** Heng Jiang, Michael Livingston, Robin Room, Anteneh Ayelign Kibret, Dallas R. English, Richard Chenhall

**Affiliations:** 1 Centre for Alcohol Policy Research, School of Psychology and Public Health, La Trobe University, Melbourne, VIC Australia; 2 Department of Public Health, School of Psychology and Public Health, La Trobe University, Melbourne, VIC Australia; 3 Centre for Health Equity, Melbourne School of Population and Global Health, University of Melbourne, Melbourne, VIC Australia; 4 National Drug Research Institute, Curtin University, Melbourne, VIC Australia; 5 Centre for Social Research on Alcohol and Drugs, Department of Public Health Sciences, Stockholm University, Stockholm, Sweden; 6 Department of Human Anatomy, School of Medicine, College of Medicine and Health Sciences, University of Gondar, Gondar, Ethiopia; 7 Centre for Epidemiology and Biostatistics, Melbourne School of Population and Global Health, University of Melbourne, Melbourne, VIC Australia; 8 Cancer Epidemiology Division, Cancer Council Victoria, Melbourne, VIC Australia

**Keywords:** Risk factors, Cancer

## Abstract

**Background:**

Long-term alcohol use is a recognised risk factor for liver, upper aerodigestive tract (UADT), colorectal, and female breast cancers, yet aggregate-level evidence linking alcohol consumption to mortality from these cancers remains limited. This study examined the potential preventive impact of reducing population drinking on cancer mortality in Australia, accounting for tobacco use and health expenditure.

**Methods:**

Annual per capita alcohol and tobacco consumption data (aged 15+) from 1910–2018, and mortality data for UADT, liver, breast, and colorectal cancers from the 1950s–2018, were collected from national registries. Time series models were used to estimate sex- and age-specific associations and long-term lagged effects of alcohol and tobacco consumption.

**Results:**

A one-litre per capita annual reduction in alcohol consumption was significantly associated with decreases in mortality: 3.6% (95% CI: 1.0–6.2%) in male and 3.4% (1.8–4.9%) in female UADT cancer; 3.9% (0.2–7.7%) in male liver cancer; 1.2% (0.7–1.7%) in male and 0.7% (0.2–1.4%) in female colorectal cancer; and 2.3% (1.7–3.0%) in female breast cancer over a 20-year period.

**Conclusion:**

Reducing population-level alcohol consumption in Australia could substantially lower mortality from UADT, colorectal, male liver, and female breast cancers, particularly among older adults.

## Introduction

Evidence from a multitude of cohort and case-control studies has led the International Agency for Research on Cancer (IARC) to conclude that alcohol consumption is a causal factor for cancers in several areas of the body. These include the oral cavity, pharynx, larynx, oesophagus, colorectum, liver (specifically hepatocellular carcinoma), and female breast [1, 2]. In meta-analyses conducted by the World Cancer Research Fund, the relative risks per 10 g/d of alcohol consumed ranged from 1.04 for liver cancer to 1.25 for oesophageal cancer [3]. While individual-level studies are essential for inferring causality, ecological studies examining the relationship between population-level consumption and cancer mortality are important for assessing the impact of public health policies. As many of alcohol or public health policy levers available to government are aimed at the general population (e.g. taxation, pricing, advertising, availability restrictions), it is crucial for policy-makers to assess how changes in population drinking relate to cancer mortality. However, associations at the aggregate level of alcohol consumption and cancer mortality have rarely been examined. There is evidence for overall cancer that changing per-capita consumption can affect mortality [4], but associations between population alcohol consumption and different cancer subtypes may vary, and there has been little research focussing on specific alcohol-related cancers.

A key consideration in understanding the relationship between alcohol consumption and cancer mortality is not only the overall level of drinking but also how alcohol consumption is distributed across a population. The collectivity theory of drinking culture, first proposed by Skog in 1985, suggests that changes in per capita alcohol consumption tend to be accompanied by shifts in drinking patterns across the entire population [5]. Specifically, when average alcohol consumption increases or decreases, the prevalence of heavy drinking also shifts in the same direction. This is critical from a public health perspective because the greatest harms, including cancer mortality, are disproportionately driven by the heaviest drinkers. Consequently, a reduction in overall alcohol consumption is not just a reflection of fewer people drinking moderately; rather, it signals a broader population-level shift that includes declines in the most harmful drinking behaviours. Studies have shown that policies aimed at reducing per capita alcohol consumption—such as taxation and restrictions on availability—have led to decreases in both mean consumption and the proportion of high-risk drinkers [6]. These findings suggest that changes in the distribution of alcohol consumption are a crucial mechanism through which population-level drinking patterns influence long-term cancer mortality trends.

Two Australian studies used historical time series data to examine the co-movement of trends in per capita alcohol consumption and trends in female breast cancer incidence [7] and cancers of the upper alimentary tract [8]. One study used lagged data and examined the temporal lags between alcohol sales and cancer mortality in 17 countries, and significant lags between alcohol sales and cancers of the lip, oral cavity and pharynx, larynx, and oesophagus were found in a range from 1 to 15 years [9]. A study examining alcohol’s role in the Australian burden of disease in 2010 estimated that 30% of male and 8% of female oral cavity and pharynx cancer deaths were alcohol-related, and attributed 15% of male and 7% of female liver cancer deaths to alcohol [10]. Similarly, an Australian cancer epidemiology study suggested that alcohol was responsible for 38% of male and 13% of female oral cavity and pharynx cancer deaths, and 16% of male and 5% of female liver cancer deaths [11]. To our knowledge, there has been no previous estimation of alcohol-attributable fractions for cancers based on historical aggregate data. In a methodological paper of aggregate analyses on alcohol and mortality, Norström and Skog have argued [12] that such aggregate-level analysis is a useful convergent-validity test of individual-level findings, and that the findings also relate more directly to the population level at which public health policies to reduce harmful effects of alcohol primarily operate.

Ecological studies of alcohol and cancer are subject to confounding by tobacco, which is a risk factor for all alcohol-related cancers except for female breast cancer. Further, for cancers of the upper aerodigestive tract (i.e., oral cavity, pharynx, larynx, and oesophagus; UADT), tobacco modifies the effect of alcohol [13], most likely because salivary acetaldehyde levels are substantially higher for smokers than non-smokers following alcohol ingestion [14]. Previous studies have also found that higher health expenditure or healthcare costs are associated with better cancer outcomes [15, 16]. Thus, ecological studies of changing alcohol consumption and cancer mortality need to carefully account for changes in tobacco consumption and health expenditure.

Another complicating factor is that there are relatively long lags between alcohol and tobacco consumption and cancer death, making aggregate time-series analyses difficult [17]. Therefore, accurately identifying the lag length between alcohol and tobacco consumption and cancer mortality is crucial for aggregated-level analysis, as ecological models can be sensitive to different lag structures. A pooled epidemiological study revealed that more than 20 years after ceasing alcohol consumption, the risks for both oesophageal and head and neck cancers were no longer significantly different from those of never drinkers [18]. Similarly, epidemiological and case-control studies found that approximately 20 years after quitting smoking, the risks for pancreatic and head and neck cancers were comparable to those of nonsmokers [19, 20]. These findings suggest a 20-year lag between alcohol and tobacco consumption and cancer mortality. Additionally, our previous studies compared various lag lengths and identified significant lagged effects of alcohol consumption on total cancer mortality in Australia over a period of 20 years [4, 21]. Using the same method, this study will further examine the lag lengths of alcohol consumption on sex-specific cancer mortality.

This study aims to estimate the magnitude of the effect of a reduction in population drinking on mortality rates from four cancer types, and how this differs across sex and age groups in Australia between the 1950s and 2018, controlling for changes in tobacco smoking and health expenditure. Furthermore, calculating AAFs using long-term historical data could provide a population-level estimate of the cumulative impact of alcohol consumption on cancer mortality over time. While previous studies have estimated AAFs using cross-sectional or short-term cohort data, our approach offers a unique temporal perspective, capturing the long-term contribution of alcohol to cancer deaths at the national level across nearly seven decades. These estimated historical AAFs are particularly relevant for informing public health planning and evaluating the potential preventive impact of population-wide alcohol policy interventions. Two research questions are addressed with respect to cancer mortality:

1. What is the association between per-capita alcohol consumption and age- and sex-specific UADT, liver and colorectal cancer, and age-specific female breast cancer?
2. What are the alcohol-attributable-fractions between per-capita alcohol consumption and mortality from different types of cancer?

## Data and method

### Time series data

A proxy for annual per-capita alcohol consumption was constructed using data on the sale of alcohol sourced from the Australian Bureau of Statistics (ABS). Alcohol consumption data between 1961 and 2018 was published officially as consumption per capita by the ABS [22], while data from earlier years (1910–1960) were extracted manually from the relevant yearbooks (e.g. Commonwealth Bureau of Census and Statistics [23]), and converted from gallons or proof gallons to litres of pure alcohol (ie., ethanol). This was then converted to litres of pure alcohol per resident aged 15 and older, using population data provided by the Australian Institute of Health and Welfare (AIHW) [24].

Annual per capita tobacco consumption (kg per capita) from 1910 to 2018 and annual per capita health expenditure (US$ per capita and adjusted to take account of the difference in purchasing power of the national currencies) from 1935 to 2018 in Australia were obtained from Cancer Council Victoria [25] and the Organisation for Economic Co-operation and Development [26], respectively. These data were included in the model to control for the impacts of tobacco consumption and health funding on the four types of cancer mortality in Australia. Smoking is one of the major risk factors for liver, UADT, and colorectal cancer and a potential risk factor for female breast cancer [1, 2]. An increase in health expenditure per capita can reduce cancer mortality rates or increase cancer survivor rates and is used here as an indicator of the level of societal effort to extend the population’s average lifespan [27].

Age- and sex-specific annual time series data of mortality rates per 100,000 population from UADT and breast cancer from 1950 to 2018 and from colorectal cancer from 1955 to 2018, were obtained from the WHO Cancer Mortality Database [28], while liver cancer mortality data from 1968 to 2018 were obtained from the Australian Institute of Health and Welfare (AIHW) [24]. Age groups include 0-29 years, 30-49 years, 50-69 years and 70 years and older. Mortality rates were age standardised to the world standard population (Segi 1960) [29] to adjust for the effects of changes in age structure on cancer mortality in the study period. More details of cancer sites and data collection are summarised in the Appendix Table A1. There were few male breast cancer cases and thus only female breast cancer was included in our analysis. Since annual alcohol consumption data were not available for specific sex or age groups in Australia, analyses in this study were based on total level of alcohol consumption and sex- and age-specific cancer mortality.

### Statistical model

The autoregressive integrated moving average (ARIMA) modelling technique was employed to estimate the association between per-capita alcohol consumption and mortality from four types of cancer (i.e., UADT, liver, breast, and colorectal cancer). ARIMA models require stationary time series to reduce the risk of obtaining a spurious relation between two series that have common trends [30]. The Augmented Dickey-Fuller (ADF) unit root test is commonly used for testing for stationarity [31]. Furthermore, the error term (which includes explanatory variables not considered in the model) is allowed to have a temporal structure that is modelled and estimated in terms of autoregressive or moving average parameters [32]. In most cases, a differencing of the time series is sufficient to eliminate non-stationarity [33]. In this study, a semi-log ARIMA model was selected, as the risk for chronic diseases is a convex function of alcohol intake [3, 34]. The final model can be written as follows:

$$\Delta {Ln}{M}_{t}=\alpha +\beta \Delta {{WA}}_{t}+\mu {\Delta C}_{i,t}+\Delta {E}_{t}$$

where $\Delta$ is the differencing operator, ${{LnM}}_{t}$ is the natural logarithm of mortality rates from cancer diseases in Australia per 100,000 inhabitants aged 15 + , ${{WA}}_{t}$ is lag weighted per-capita alcohol consumption, ${C}_{i,t}$ are the control variables considered in the estimation, *i* is number of control variables, $\mu$ is the coefficient values of the control variables, ${E}_{t}$ is the error term including other causal factors, and $\alpha$ is the constant. The key coefficient value $\beta$ indicates the proportional change in cancer mortality rates associated with a one-litre change in weighted per-capita 15+ consumption. Therefore, a coefficient value of $\beta$ means that a one-litre increase in annual alcohol consumption per capita is associated with a relative increase in the rate of cancer mortality of about $\left({e}^{\beta }-1\right)\times 100$ and *e* ≈ 2.71828.

In this study, two control variables were included in the models: tobacco consumption and health expenditure per capita. The model fit was evaluated with the aid of the Box-Ljung portmanteau test of the first 10 autocorrelations, *Q* (10). The model structures used are reported below, alongside the output of the models. Overall, there were 28 ARIMA models fitted in the study by sex and age groups.

### Lags between alcohol and tobacco consumption and cancer mortality

**Fig. 1:**
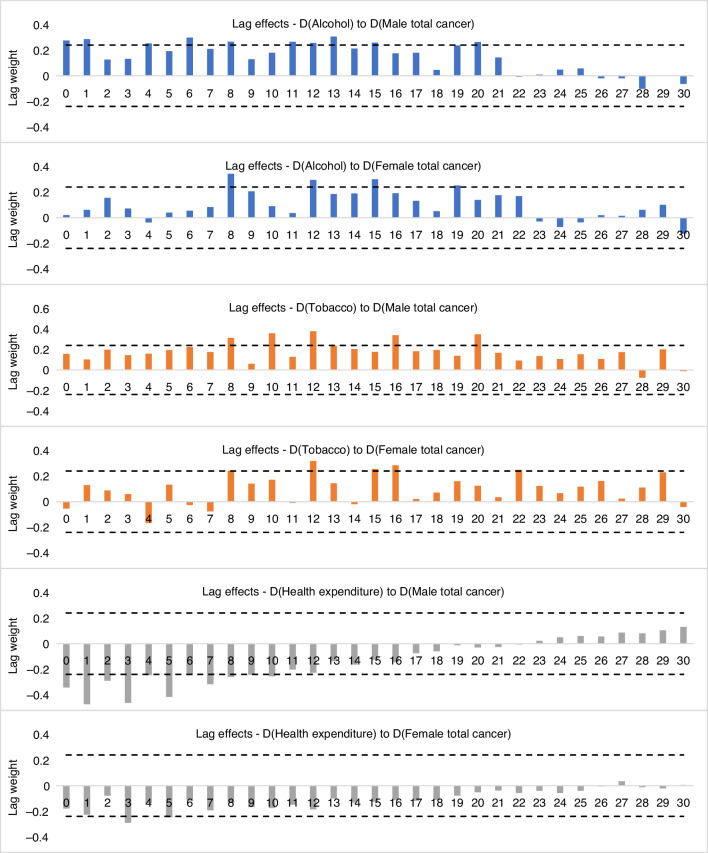
Lag length of changes in population drinking and smoking on male and female mortality rates of overall cancer. The critical values of the cross-correlation test were calculated based on |±2/$\sqrt{n}$| = 0.240 and *n* = 69 is number of years in the sample, the first-differenced data, including D(cancer), D(alcohol), D(tobacco), and D(health expenditure) were used in the analysis.

Our previous studies found that any effects on cancer mortality of changes in population drinking and smoking were not fully seen in the year in which the change occurred [4, 21]. Following methods used in our previous studies [4, 35, 36], we conducted cross-correlation tests to explore cross-correlation relationships between per capita alcohol and tobacco consumption and rates of male and female total cancer mortality in Australia to identify lag lengths of long-term alcohol and tobacco use on cancer mortality. The results of a cross-correlation test (based on the first differenced data with trends and autocorrelation removed, see Appendix for more information) between alcohol and tobacco consumption and overall male and female cancer mortality are presented. A significant and positive correlation was found between alcohol consumption and overall male and female cancer mortality, and the results suggest that 20- and 19-year lags on alcohol consumption could be applied in the male and female cancer estimation models, respectively. The cross-correlation analyses also indicate that lag lengths for the associations between smoking and overall male and female cancer mortality were 20 and 22 years, respectively. There were significant cross-correlations between health expenditure per capita and male and female total cancer mortality with lag lengths of 10 and 5 years, respectively (Fig. 1).

The estimated cross-correlation lag weights were then used to build sex-specific lagged alcohol and tobacco consumption, and health expenditure series in order to measure the relationships between alcohol and tobacco consumption and various subtypes of cancer mortalities while controlling for the preventive effects from increase in health expenditure per capita.

### Computing alcohol attributable fraction

The calculation method of alcohol attributable fraction (AAF) for cancer mortality based on aggregate data analysis has been elaborated in previous studies (e.g. Ramstedt [37] and Jiang et al. [38]), and the equation can be presented as:

$${AAF}=\frac{{{\rm{AACP}}}* {{\rm{E}}}}{{{\rm{AM}}}}$$

$$E=\left({e}^{\beta }-1\right)\times 100* {AM}$$

where AACP is the average alcohol consumption per capita during the observation period; E is the estimated annual cancer mortality rate attributable to each litre alcohol consumption and AM is the average mortality rate during the observation period; $\frac{{{\rm{E}}}}{{{\rm{AM}}}}=\left({e}^{\beta }-1\right)\times 100$, and this represents the relative or proportional increase in the cancer mortality rate attributable to a one-litre increase in per capita alcohol consumption in the study period. To estimate the expected reduction in mortality associated with a decrease in alcohol consumption (e.g., a one-litre decrease), the same logic applies in the opposite direction, assuming the relationship is symmetrical over small changes. AAF will be calculated if there are significant associations between alcohol consumption and selected cancer mortality rates. Alcohol consumption data from eight waves of Australian National Drug Strategy Household Surveys (NDSHS), spanning from the 1990s to 2019, suggest that males accounted for approximately 67.5% of the total alcohol consumption during the 1990s and in 2019 [39, 40]. This ratio was used to computing AAFs for male and female cancer morality in the following analyses.

### Sensitivity analyses

Previous studies have used 15-year and 20-year geometric lag structures to examine associations between alcohol consumption and liver diseases [17]. Therefore, this alternative lag structure was employed to test the robustness of findings to different assumptions about latency periods. This approach incorporates the lagged effects of alcohol or tobacco consumption by assigning greater weights to more recent years (shown in Appendix Fig. A1). Furthermore, health expenditure per capita was excluded in models to assess the influence on the estimated associations between per capita alcohol consumption and various cancer mortality rates. Additionally, to assess whether the observed associations were specific to alcohol-related cancers, we conducted an additional ARIMA time-series analysis using male and female lung cancer mortality as the outcome variables and cross-correlation lagged alcohol and tobacco consumption as predictors. Because epidemiological literature shows that male and female lung cancer mortality rates are predominantly associated with tobacco use and have not been consistently linked to alcohol consumption. R-squared values and confidence intervals from the main models were compared with those from the sensitivity analyses. A higher R-squared indicates that more variation in cancer mortality is explained by the lagged exposure variable, while narrower confidence intervals suggest more precise estimates.

## Results

### Trends in alcohol and tobacco consumption and four types of cancer mortality rates

**Fig. 2:**
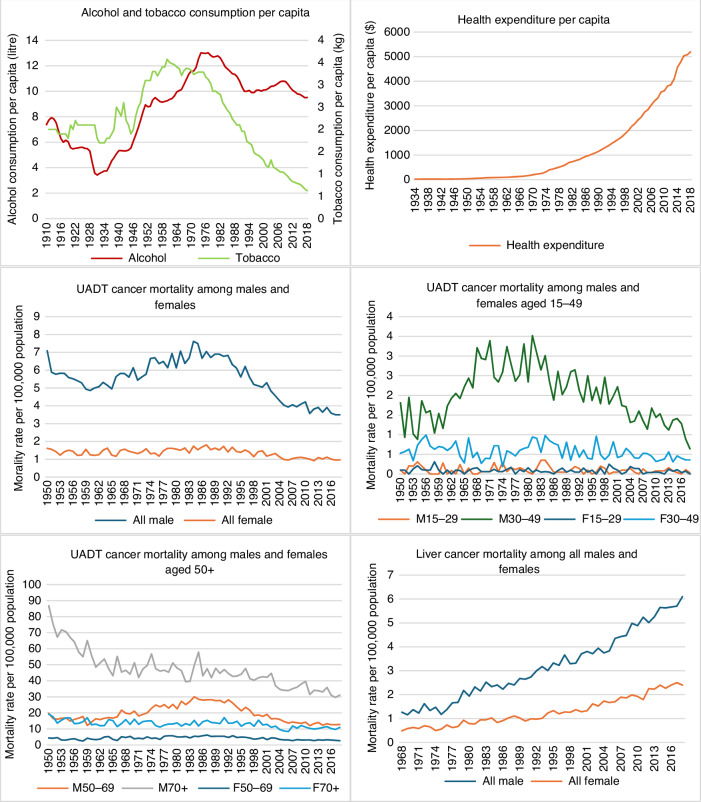

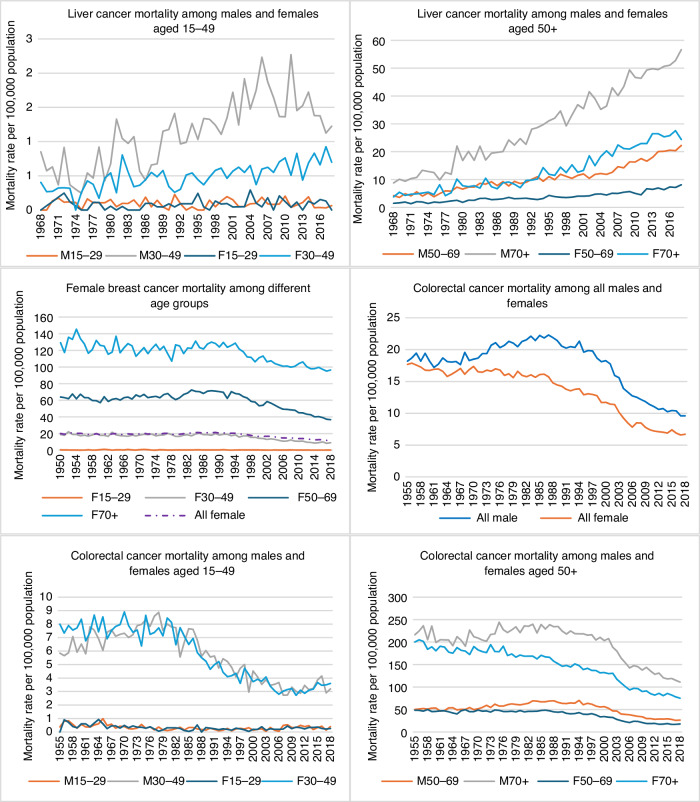
Trends in alcohol and tobacco consumption per capita (15+), health expenditure per capita, and upper aerodigestive tract—UADT (lip, oral cavity, pharynx, larynx and oesophagus) cancer, liver cancer and colorectal cancer mortality rates among males and females, and female breast cancer mortality per 100,000 population.

Trends in alcohol and tobacco consumption per capita (aged 15 and over), and per capita health expenditure and age and sex specific UADT, liver, breast, and colorectal cancer mortality rates per 100,000 population were presented in Fig. 2. Per capita alcohol consumption increased significantly between 1950 and 1970, and tobacco consumption increased between 1950 and 1966 and decreased steadily after that. Per capita health expenditure increased steadily from 1935 to 2018. There was an increase in UADT cancer mortality among males between 1960 and 1986. Male and female UADT cancer mortality rates decreased after the 1980s among people aged 50 and over. Mortality of UADT cancers among those aged 70 and over, and 50–69-year-olds, was higher than in younger age groups. The cancer mortality rates of four subtypes among the youngest male and female age groups (0-29 years old) were small, and these age groups were thus excluded from the age-specific analysis.

A steady increase in liver cancer mortality rates occurred in Australia after 1968, from 1.3 to 6.1 deaths for males and 0.5 to 2.4 deaths for females in 2018 per 100,000 population aged 15 and over. In this period, the trends in Australian alcohol and tobacco consumption per capita were divergent from the trend of liver cancer mortality rates. The female breast cancer mortality rates remained at roughly the same level from 1950 to 1995 and decreased slightly after 1995. Male colorectal cancer mortality rates broadly followed changes in per capita alcohol consumption, increasing from 1955 to 1986 and then declining after that, while female colorectal cancer mortality rates have decreased gradually since 1955.

### Temporal associations between alcohol consumption and cancer mortalities in subtypes

Age and sex-specific model outputs of the cross-correlation lagged alcohol consumption and UADT cancer mortality rates models are summarised. The results suggest that a one litre decrease in annual per capita alcohol consumption could reduce UADT cancer mortality rates for males by 3.6% [(e^0.035-1^)*100] and for females by 3.4% across a 20-year period, controlling for the effects of tobacco consumption and health expenditure per capita. Compared with other age groups, significant associations were found between per capita alcohol consumption and UADT cancer mortality rates among both males and females aged 50-69 years only (Table 1).

**Table 1:** Semi-log ARIMA models on associations of alcohol and tobacco consumption with upper aerodigestive tract (UADT) liver cancer mortality among different gender and age groups.

	Alcohol consumption			Tobacco consumption			ARIMA model term
	Estimate	95% CI	*p*- value	Estimate	95% CI	*p*- value	
Male UADT cancer							
30–49	−0.086	−0.247, 0.075	0.294	0.512	0.413, 0.611	0.000	0,1,1
50−69	0.146	0.060, 0.231	0.001	0.265	0.026, 0.504	0.030	0,1,1
70+	0.095	−0.017, 0.208	0.097	−0.092	−0.245, 0.060	0.234	0,1,1
All males	0.035	0.010, 0.060	0.007	0.241	0.184, 0.298	0.000	0,1,1
Female UADT cancer							
30–49	0.066	−0.160, 0.292	0.567	0.135	−0.043, 0.313	0.136	0,1,1
50–69	0.122	0.009, 0.155	0.000	0.276	0.116, 0.435	0.001	0,1,1
70+	0.026	−0.019, 0.071	0.259	0.023	−0.115, 0.160	0.748	0,1,1
All females	0.033	0.018, 0.048	0.000	0.143	0.012, 0.274	0.032	0,1,1
Male liver cancer							
30–49	0.002	−0.152, 0.157	0.976	−0.121	−0.688, 0.444	0.673	1,1,1
50–69	0.074	0.035, 0.144	0.040	0.231	−0.034, 0.496	0.087	0,1,1
70+	0.071	0.028, 0.113	0.001	0.280	0.071, 0.489	0.009	1,1,3
All males	0.038	0.002, 0.074	0.041	0.211	0.102, 0.319	0.000	0,1,1
Female liver cancer							
30–49	0.010	−0.110, 0.090	0.840	−0.740	−0.571, 0.423	0.770	0,1,1
50–69	0.030	−0.005, 0.066	0.091	0.092	−0.080, 0.264	0.294	0,1,1
70+	−0.030	−0.082, 0.022	0.265	−0.073	−0.248, 0.101	0.411	0,1,1
All females	−0.007	−0.050, 0.036	0.740	−0.025	−0.125, 0.757	0.631	0,1,1

The results of temporal associations between lagged alcohol consumption and liver cancer mortality rates in males and females are summarised. It depicts a significant association between alcohol consumption per capita and liver cancer mortality for males, but not for females. The results suggest that a 1 L decrease in per capita alcohol consumption could reduce male liver cancer mortality rates by 3.8%. There is a significant positive association between alcohol consumption and liver cancer mortality rates only among the middle and older male age groups (aged 50+ years), with no associations found in younger males (aged 30–49 years).

The results of time series analyses reveal that there were positive associations between colorectal cancer mortality rates and per capita alcohol consumption among groups of aged 50 years and over in both males and females (Table 2). A one litre decrease in per capita alcohol consumption was associated with lower male and female colorectal cancer mortality rates: 1.2% and 0.7% respectively. From the age-specific model outputs of lagged alcohol consumption with female breast cancer mortality rates, a one litre decrease in per capita alcohol consumption was associated with a 2.3% [(e^0.023–1^)*100] reduction in female breast cancer mortality rates. Positive statistically significant associations between alcohol consumption and female breast cancer mortality rates were found across all age groups (30+ years).

**Table 2:** Semi-log ARIMA models on associations of alcohol and tobacco consumption with colorectal cancer and female breast cancer mortality among different gender and age groups.

	Alcohol consumption			Tobacco consumption			ARIMA model term
	Estimate	95% CI	*p*- value	Estimate	95% CI	*p*- value	
Male colorectal cancer							
30–49	−0.018	−0.204, 0.169	0.854	0.039	−0.014, 0.092	0.147	0,1,1
50–69	0.014	0.007, 0.021	0.000	0.030	0.009, 0.051	0.005	0,1,1
70+	0.009	0.004, 0.015	0.001	0.017	0.002, 0.031	0.022	0,1,1
All males	0.012	0.007, 0.017	0.000	0.024	0.010, 0.038	0.001	0,1,1
Female colorectal cancer							
30–49	−0.001	−0.131, 0.129	0.991	0.024	−0.012, 0.059	0.191	0,1,1
50–69	0.011	0.005, 0.017	0.000	0.030	0.013, 0.046	0.000	0,1,1
70+	0.005	0.001, 0.010	0.024	0.015	0.004, 0.026	0.006	0,1,1
All females	0.007	0.002, 0.014	0.013	0.024	0.007, 0.040	0.005	0,1,1
Female breast cancer							
30–49	0.026	0.016, 0.037	0.000	0.016	−0.003, 0.036	0.509	0,1,1
50–69	0.032	0.024, 0.041	0.000	0.030	0.013, 0.046	0.000	0,1,1
70+	0.030	0.001, 0.586	0.041	0.045	−0.044, 0.134	0.319	0,1,1
All females	0.023	0.017, 0.030	0.000	0.064	−0.114, 0.240	0.485	0,1,1

Assuming that 67.5% of the total alcohol was consumed by males based on the published reports of the NDSHS the estimated average alcohol consumption per capita was 12.6 and 6.1 per litre among males and females during 1930 and 2018. These suggest that between the 1950s and 2018, about 45.4% (3.6%*12.6) of male and 20.7% (3.4%*6.1) of female UADT cancer deaths, 47.8% (3.8%*12.6) of male liver cancer deaths were alcohol−related.

The results of sensitivity analyses are summarised in Tables A2–A5 in Appendix. Models using the geometric lag structures yielded no statistically significant associations between alcohol consumption and subtypes of cancer mortality rate, suggesting a more conservative effect estimate under these alternative lag assumptions. Excluding health expenditure from the models produced stronger positive associations, particularly for liver and breast cancer. Comparison of R-squared and confidence interval values between the main models and sensitivity analyses shows that models using cross-correlation lag structures, while controlling for health expenditure, produced higher R-squared values and tighter confidence intervals than those using geometric lag structures or models that did not control for health expenditure (please see Appendix Tables A2–A4 for details). The sensitivity analysis of male and female lung cancer mortality and cross-correlation lagged alcohol and tobacco consumption showed that lagged alcohol consumption was not significantly associated with lung cancer mortality in either males or females, whereas lagged tobacco consumption demonstrated the expected strong and significant positive associations (Table A5 in Appendix).

## Discussion

The study provides strong research evidence from an aggregate-level analysis that a decrease in population-level drinking could lead to a reduction in UADT cancer, colorectal cancer and male liver cancer mortality rates in Australia, particularly among people aged 50–69. Decreasing alcohol consumption may also reduce female breast cancer mortality rates across all three age groups—30–49, 50–69 and 70+ years. It is worth noting that the main results in the study are highly sensitive to the choice of lag structure. This sensitivity suggests a degree of uncertainty around the estimated associations and reinforces the need for cautious interpretation.

There are thus similar results between our aggregate analyses and previous epidemiological and burden of disease studies on alcohol and UADT cancer (or oral cavity and pharynx cancer), by which about 38–45% of male and 13–17% of female UADT cancer incidents were attributable to alcohol use [11, 41, 42]. Our results suggest that reducing alcohol consumption at the population level may have higher preventive impacts on UADT cancer mortality for males than for females. In substantial part, this no doubt reflects that males account for considerably more than females of the total alcohol consumption.

Our analyses suggest that, with respect to liver cancer, reducing alcohol consumption per capita might only reduce male mortality, with no effect on female liver cancer. These findings are consistent with the epidemiological literature [43, 44], which suggests a higher alcohol-attributed fraction for male liver cancer mortality. Our estimation of alcohol attributable fraction for male liver cancer deaths is higher than a previous Australian estimate in which Pandeya et al. estimated that [11] 16% of male liver cancer deaths in 2010 were attributable to alcohol. A possible reason is that the rates of Hepatitis C and B viruses infection increased significantly in the 1980s in Australia and particularly among Asian population, and the effect of alcohol consumption on liver cancer mortality might possibly *increase* with higher rates of HBV and HCV [45, 46]. This is mainly because alcohol and HBV/HCV infections synergistically contribute to liver damage, exacerbating inflammation and fibrosis, which accelerates progression to cirrhosis and increases liver cancer risk [47]. Furthermore, chronic alcohol use impairs the immune system, allowing viral infections to persist and cause more extensive damage [48]. Additionally, alcohol can increase HBV and HCV replication and mutagenesis, leading to more aggressive viral forms and a heightened risk of liver cancer [49]. Unfortunately, long-term time series data on the HBV and HCV rates in Australia were not available, and we are unable to control for the effects of HBV and HCV, and other factors that may affect liver cancer mortality, such as new drugs for liver cancer treatment in the study period.

Additionally, we found positive associations between alcohol consumption and colorectal cancer mortality rates, revealing that 15.1% of male and 4.3% female colorectal cancer deaths were attributable to alcohol consumption in Australia. This is in line with results in previous epidemiological studies [3, 42, 50]. These results indicate that a reduction of one litre per capita in alcohol consumption was linked to a decrease in male and female colorectal cancer mortality rates, amounting to 1.2% and 0.7%, respectively.

The associations between alcohol consumption per capita and liver, UADT and colorectal cancer mortality rates were found only among older age groups, particularly among those aged 50 and over. This can be seen as reflecting the long-term effects of alcohol consumption on the development of malignant neoplasms in the human body. Using data from the National Drug Strategy Household Surveys between the 1980s and 2016, we found that younger age groups reduced their alcohol consumption in the last 30 years, while the drinking levels of older groups remained the same in Australia [51]. The ageing population in Australia may lead to a greater number of alcohol-related cancer deaths among older age groups in the future, if per capita alcohol consumption remains at the same level. Furthermore, positive but weaker associations between alcohol consumption per capita and liver, UADT and colorectal cancer mortality rates were found among the group aged 70 and over. In this age group, competing causes of death, such as from other chronic diseases and injuries from falls, may reduce the toll from these cancers [38, 52].

A significant association between alcohol consumption per capita and female breast cancer mortality rates was found in this study, suggesting that reducing alcohol consumption will reduce or prevent female breast cancer incidence across nearly all age groups in females in Australia. This result is consistent with previous epidemiological evidence. But while a previous Australian epidemiological study suggested that 6% of breast cancer incidence could be associated with total alcohol consumption [42], we found a higher alcohol attributable fraction for female breast cancer mortality (14%). This difference may reflect several factors: first, alcohol may contribute more to mortality than incidence due to its role in more aggressive cancer types or delayed detection. Second, our use of long-term, population-level exposure data with lag structures captures cumulative risk, whereas earlier studies may rely on short-term self-reported intake. Third, by adjusting for tobacco use and health expenditure, our model may more accurately isolate alcohol’s effect, reducing residual confounding. Together, these factors suggest that prior estimates may have underestimated the long-term impact of alcohol on breast cancer outcomes.

The revised Australian Guidelines to Reduce Health Risk from Drinking Alcohol [53] released by the National Health and Medical Research Council in December 2020 state that males and females should drink no more than 10 standard drinks a week and no more than 4 standard drinks on any one day to reduce the lifetime risk of harm attributed to alcohol, such as cancer, cardiovascular diseases and mental disorders. If more of the population followed these drinking guidelines, this would reduce the risk of developing alcohol-related cancers substantially. Furthermore, the WHO states that there is no level of alcohol consumption which is safe for risk of cancer [54]. Our study findings also revealed that one litre decreases in annual alcohol consumption per capita were associated with reductions of 3.6% in male and 3.4% in female UADT cancer mortality, reductions of 3.9% in male liver cancer mortality, reductions of 1.2% in male and 0.7% in female colorectal cancer mortality and 2.3% female breast cancer mortality across a 20-year period in Australia. Additionally, public health policies on alcohol that recommended by the WHO as “best buys” [55], such as reducing affordability by increasing alcohol prices or tax, increasing control of alcohol availability, providing brief interventions for heavy drinkers to moderate their drinking, and restricting on exposure to alcohol advertising, are among the means by which the toll of cancer diseases can be reduced in Australia.

### Limitations

We use overall alcohol and tobacco consumption per person aged 15 and over in the estimation, since no age or sex-specific annual consumption per capita data is available in Australia. This could affect our estimates of how much sex or age-specific cancer mortality could be reduced by a decrease in overall alcohol consumption, especially where there have been changes in the relative contribution to total consumption of different age and sex sub-groups. For example, smoking rate peaked in 1950s for males, which was much earlier than females. Besides alcohol and tobacco consumption, there are many other factors which could affect rates of UADT cancer, liver cancer, and breast cancer, such as infectious diseases (for example, HPV, HBV, HCV), insufficient physical activity and diet, and these factors were not considered in our study. Nevertheless, the use of differenced data in the ARIMA models reduces the risk of this kind of confounding, unless change in the unmeasured variable is correlated with change in both per-capita alcohol consumption and cancer mortality. This may still be an issue for some confounds, such as overweight and obesity. Unfortunately, long-term historical obesity rate data are not available in Australia, and we were unable to include this variable in our models. Additionally, life expectancy, overall mortality rates, or mortality from non-alcohol-related cancers could also serve as potential indicators of general population health or competing risks. However, we aimed to avoid over-adjustment and multicollinearity by selecting a limited number of covariates that were both theoretically justified and available across the full time series.

The multiplicative relationship between alcohol and tobacco consumption has been observed in individual-level cancer studies. However, this multiplicative effect cannot be estimated in our time series model because the time series analysis does not allow for interaction estimation. The effects of the patterning of the drinking— on dimensions such as frequency of drinking occasions and whether there is heavy episodic drinking—or the magnitude of dose-response effects cannot, of course, be estimated from our aggregate analyses. It should be noted that the analyses presented here use innovative lag-structure approaches to attempt to account for the temporal delays between alcohol consumption and cancer mortality. The associations observed using 20-year cross-correlation lags support the importance of long-term cumulative exposure. The lags selection was based on total cancer mortality, and we assume no difference in time delays between drinking and different cancer types.

We conducted a series of sensitivity analyses to examine the associations between alcohol and tobacco consumption and various cancer mortality subtypes in males and females, using either a 20-year or 15-year geometric lag structure, or without controlling for the effects of increasing health expenditure per capita. No associations were found between per capita alcohol consumption and the various types of cancer mortality in either males or females in the time series models with the 20-year or 15-year geometric lag structure. The lack of significant results under geometric lags may reflect the smoothed weighting favouring recent consumption, which may underestimate the long-term carcinogenic effects of alcohol. However, the effects of alcohol consumption on four cancer mortality subtypes were greater in models that did not control for health expenditure, meaning that health expenditure may confound the association of interest and that its inclusion in the model helps isolate the specific contribution of alcohol consumption to cancer mortality trends. The stronger effects when excluding health expenditure suggest that improvements in health services may mitigate some alcohol-related cancer harms, supporting the role of broader system-level factors. This result may also imply that increased health expenditure also likely resulted in increased early detection and better cancer treatment, thus reducing cancer mortality despite the related alcohol harms.

These sensitivity results do not undermine the primary findings but instead highlight the robustness of the main conclusions, particularly the consistent direction of associations across modelling approaches. The findings from the additional sensitivity analysis of male and female lung cancer mortality reinforce the specificity of the associations observed in our primary models, supporting the interpretation that the effects of alcohol consumption are concentrated in alcohol-related cancer types rather than reflecting a general trend across all cancer mortality. Our previous studies [4, 21] are consistent with the current study in that they revealed that using cross-correlation lag structures to examine the associations between per capita alcohol and tobacco consumption and cancer mortality outperformed other lag structures, such as the geometric lag structure. The absence of significant associations under geometric lags may suggest that shorter-term changes in alcohol use and assigning heavier weights to recent exposure/years may not fully capture the latency-related effects. Nevertheless, the fact that the associations between alcohol and various cancer mortalities were non-significant using alternative lag structures raises some concerns about the robustness of these findings, and future work is necessary to develop the appropriate ways to estimate the complex lagged effects of alcohol and tobacco on cancer mortality outcomes.

The results, while indicative of a potential long-term association between alcohol consumption and cancer risk, are not robust across different lag assumptions and should be interpreted with caution. More research is needed to confirm the effects identified here, including the temporal causal relationships between long-term alcohol consumption and mortality rates in different cancer subtypes. But it may be noted that overall, the findings from these aggregate time series analyses are convergent with the results from individual-level longitudinal studies. The policy implications of the study are that there would be significant preventive effects on UADT, colorectal, male liver, and female breast cancer deaths, particularly among older age groups, from reducing population drinking levels.

## Supplementary information

Appendix

## Data Availability

All data used in this study are publicly available and can be obtained from the WHO, OECD and Australian data agencies. Mortality rates for four cancer subtypes are publicly available at WHO Cancer Mortality Database (URL: https://gco.iarc.fr/overtime/en). A longer term of mortality rate for liver cancer can be obtained from the Australian Cancer Database at the Australian Institute of Health and Welfare (AIHW) (URL: https://www.aihw.gov.au/reports/cancer/cancer-data-in-australia/data). Please note that the Australian Cancer Mortality data in WHO Cancer Mortality Database were provided by AIHW. Annual alcohol consumption per capita data are available at Australian Bureau of Statistics (ABS) (URL: https://www.abs.gov.au/statistics/health/health-conditions-and-risks/apparent-consumption-alcohol-australia/latest-release#:~:text=Per%20capita%20consumption,of%20around%201.1%25%20per%20year). Annual tobacco consumption per capita data can be collected from Tobacco in Australia: Fact and issues website published by Cancer Council Australia (URL: https://www.tobaccoinaustralia.org.au/chapter-2-consumption). Data of health expenditure per capita are available at OECD Health Statistics Database (URL: https://data-explorer.oecd.org/).

## Code availability

Stata version 18 was used to analyse all time series models and data in this study. Stata syntax can be provided upon request.
