# Supplementary material for: Alcohol consumption and mortality from four alcohol-related cancers in Australia 1950-2018: a time series analysis
Source: Br J Cancer. 2026 Jan 9;134(6):914–23. doi: 10.1038/s41416-025-03273-1 (PMC12960789; doi:10.1038/s41416-025-03273-1)
Supplement: Supplementary file 1 — Appendix [file 41416_2025_3273_MOESM1_ESM.docx]

**Appendix**

**Table A1** Description of four types of cancer mortality data

| **Name and ICD-10 codes** | **Description** | **Time period** | **Source** |
| --- | --- | --- | --- |
| Upper aerodigestive tract (UADT) cancer; C00–C14, C32, | Malignant neoplasms of lip, oral cavity and pharynx;  Malignant neoplasms of oesophagus;  Malignant neoplasm of larynx | 1950-2018 | WHO Cancer Mortality Database |
| Liver cancer; C22 | Malignant neoplasm of liver and intrahepatic bile ducts | 1968-2018 | AIHW Cancer Mortality Database |
| Breast cancer; C50 | Malignant neoplasm of breast | 1950-2018 | WHO Cancer Mortality Database |
| Colorectal cancer; C18-20 | Malignant neoplasm of colon;  Malignant neoplasm of rectosigmoid junction;  Malignant neoplasm of rectum; | 1955-2018 | WHO Cancer Mortality Database |

Note: Australian liver cancer mortality data were also available in the WHO cancer mortality database, but with a shorter time period (1980-2018). The data stored in the WHO cancer mortality database were provided by the Australian Institute of Health and Welfare (AIHW), and we abstracted the liver cancer mortality data (1968-2018) from the AIHW.

**Stationary**

In time series analysis, the term "stationary" refers to a property of a time series where its statistical characteristics—such as the mean, variance, and autocorrelation—do not change over time. A stationary time series is essential for many time series models because it ensures that the underlying patterns are consistent and predictable, allowing for more reliable modelling and forecasting.

The term "first differenced data" refers to a transformation applied to a time series to make it more suitable for analysis, particularly when the original data is non-stationary, meaning that its statistical properties (like mean and variance) change over time.

The first difference of this time series is obtained by subtracting the previous value from the current value. It can be expressed as $D(first difference)=X_{t}-X_{t-1}$

**Cross-correlation test**

The cross-correlation test in time series analysis is a statistical tool used to measure the relationship between two time series. It helps to determine whether one time series is related to another, and if so, to identify the time lag at which this relationship is strongest. Please refer to this article about the cross-correlation test

**Sensitivity analyses**

Sensitivity analyses were conducted to examine the associations between alcohol and tobacco consumption and various cancer mortality subtypes in males and females, using either a 20-year or 15-year geometric lag structure (Tables A2 and A3), or without controlling for the effects of increasing health expenditure per capita (Table A4). No associations were found between per capita alcohol consumption and the various types of cancer mortality in either males or females in the time series models with the 20-year or 15-year geometric lag structure. Similar results were observed in the models that did not include health expenditure per capita as a covariate. However, the effects of alcohol consumption on four cancer mortality subtypes were greater in models that did not control for health expenditure.

From a public health perspective, the population's drinking level needs to be reduced by one litre following the introduction of a policy or intervention over many years. The impact of a one-litre reduction in alcohol consumption for 20 years would be fully observed over a 20- to 40-year period after the reduction.

**Table A2** A sensitivity analysis using 20 years geometric lag structure

|  | Alcohol consumption | | | Tobacco consumption | | | ARIMA model term |
| --- | --- | --- | --- | --- | --- | --- | --- |
|  | Estimate | 95% CI | p-value | Estimate | 95% CI | p-value |  |
| UADT cancer |  |  |  |  |  |  |  |
| All males | 0.028 | -0.036, 0.091 | 0.391 | -0.333 | -0.542, -0.124 | 0.002 | 1,1,3 |
| All females | 0.005 | -0.082, 0.091 | 0.918 | -0.077 | -0.309, 0.155 | 0.514 | 1,1,1 |
| Liver cancer |  |  |  |  |  |  |  |
| All males | -0.002 | -0.293, 0.289 | 0.992 | -0.201 | -1.648, 1.246 | 0.785 | 1,1,1 |
| All females | 0.102 | -0.081, 0.285 | 0.274 | -0.467 | -1.573, 0.639 | 0.408 | 1,1,1 |
| Colorectal cancer |  |  |  |  |  |  |  |
| All males | 0.004 | -0.061, 0.070 | 0.895 | -0.033 | -0.237, 0.171 | 0.752 | 0,1,1 |
| All females | 0.013 | -0.095, 0.121 | 0.816 | -0.045 | -0.464, 0.374 | 0.835 | 2,1,1 |
| Female breast cancer | -0.002 | -0.052, 0.048 | 0.935 | -0.018 | -0.145, 0.110 | 0.787 | 1,1,1 |

**Table A3** A sensitivity analysis using 15 years geometric lag structure

|  | Alcohol consumption | | | Tobacco consumption | | | ARIMA model term |
| --- | --- | --- | --- | --- | --- | --- | --- |
|  | Estimate | 95% CI | p-value | Estimate | 95% CI | p-value |  |
| UADT cancer |  |  |  |  |  |  |  |
| All males | 0.032 | -0.045, 0.109 | 0.414 | -0.318 | -0.570, -0.066 | 0.013 | 1,1,3 |
| All females | 0.001 | -0.083, 0.086 | 0.977 | -0.016 | -0.314, 0.281 | 0.915 | 0,1,1 |
| Liver cancer |  |  |  |  |  |  |  |
| All males | 0.022 | -0.224, 0.269 | 0.960 | 0.316 | -1.607, 0.975 | 0.632 | 1,1,1 |
| All females | 0.094 | -0.066, 0.254 | 0.248 | -0.443 | -1.455, 0.569 | 0.391 | 1,1,1 |
| Colorectal cancer |  |  |  |  |  |  |  |
| All males | 0.010 | -0.053, 0.073 | 0.745 | -0.062 | -0.261, 0.137 | 0.540 | 1,1,1 |
| All females | -0.001 | -0.109, 0.107 | 0.986 | -0.017 | -0.494, 0.461 | 0.945 | 2,1,1 |
| Female breast cancer | 0.000 | -0.051, 0.052 | 0.990 | -0.052 | -0.199, 0.095 | 0.487 | 1,1,1 |

**Table A4** A sensitivity analysis using cross-correlation lag structure with excluding of health expenditure per capita

|  | Alcohol consumption | | | Tobacco consumption | | | ARIMA model term |
| --- | --- | --- | --- | --- | --- | --- | --- |
|  | Estimate | 95% CI | p-value | Estimate | 95% CI | p-value |  |
| UADT cancer |  |  |  |  |  |  |  |
| All males | 0.085 | 0.013, 0.157 | 0.021 | 0.152 | -0.057, 0.362 | 0.154 | 0,1,1 |
| All females | 0.071 | 0.035, 0.107 | 0.000 | 0.149 | -0.015, 0.283 | 0.030 | 1,1,1 |
| Liver cancer |  |  |  |  |  |  |  |
| All males | 0.047 | 0.019, 0.075 | 0.001 | 0.160 | 0.074, 0.246 | 0.000 | 0,1,1 |
| All females | -0.007 | -0.047, 0.033 | 0.731 | -0.023 | -0.121, 0.076 | 0.652 | 0,1,1 |
| Colorectal cancer |  |  |  |  |  |  |  |
| All males | 0.012 | 0.007, 0.017 | 0.000 | 0.0245 | 0.011, 0.039 | 0.001 | 0,1,1 |
| All females | 0.009 | 0.002, 0.016 | 0.009 | 0.022 | 0.004, 0.041 | 0.019 | 0,1,1 |
| Female breast cancer | 0.067 | 0.036, 0.097 | 0.000 | 0.152 | 0.057, 0.247 | 0.002 | 0,1,1 |

**Table A5** A sensitivity analysis using male and female lung cancer mortality rate per 100,000 population as outcomes and cross-correlation lagged alcohol and tobacco consumption as predictors

| Log (male lung cancer mortality rate) with ARIMA (1,1,1) | Coefficient | 95% CI lower | 95% CI Upper | P>z |
| --- | --- | --- | --- | --- |
| Lag alcohol consumption | 0.067 | -0.054 | 0.188 | 0.277 |
| Lag tobacco consumption | 0.482 | 0.196 | 0.768 | 0.001 |
|  |  |  |  |  |
| Log (female lung cancer mortality rate) with ARIMA (0,1,1) | Coefficient | 95% CI lower | 95% CI Upper | P>z |
| Lag alcohol consumption | 0.044 | -0.052 | 0.142 | 0.366 |
| Lag tobacco consumption | 0.291 | 0.127 | 0.453 | 0.000 |

**Geometric lag weight**

A geometrical lag scheme was used in the estimation with λ=0.7. This approach builds in the lagged effects of alcohol or tobacco consumption, with higher weights placed on more recent years (shown in Figures A1) [1].

Geometric weight = $\frac{X_{n}+{0.7}^{2}X_{n-1}+\ldots+{0.7}^{n-1}X_{2}+{0.7}^{n}X_{1}}{1+0.7+{0.7}^{2}+\ldots+{0.7}^{n-1}+{0.7}^{n}}$

**Figure A1** Geometric distribution of lag effects of alcohol or tobacco consumption on cancer mortality in the past 15 and 20 years

**References**

1. Jiang H, Livingston M, Room R, Dietze P, Norström T, Kerr WC: Alcohol consumption and liver disease in Australia: A time series analysis of the period 1935–2006. Alcohol and Alcoholism 2013, 94(3):363-368.
